# Is cardiac MRI adenosine stress perfusion imaging a more appropriate diagnostic tool for obese patients?

**DOI:** 10.1186/1532-429X-14-S1-P76

**Published:** 2012-02-01

**Authors:** Ronald J Mikolich, John Lisko, Brandon M Mikolich

**Affiliations:** 1 Northeast Ohio Medical University, Rootstown, OH, USA; 2 Sharon Regional Health System, Hermitage, PA, USA

## Background

Cardiac nuclear stress perfusion imaging is subject to artifact related to obesity, breast attenuation and diaphragm overlap. These factors are responsible for “false positive” results among patients undergoing evaluation for coronary atherosclerosis, leading to potentially unnecessary invasive coronary arteriography. Cardiac MRI adenosine stress perfusion imaging is unaffected by both obesity and breast tissue attenuation, resulting in sensitivity and specificity rates in excess of 90%. Accordingly, cardiac MRI adenosine stress perfusion imaging may be a more appropriate form of testing for obese patients.

## Methods

Interrogation of the institutional cardiac MRI database revealed 110 patients who had undergone both cardiac nuclear stress perfusion imaging and cardiac MRI adenosine stress perfusion imaging studies, separated in time by no more than 3 months. Each type of perfusion imaging study was categorized as Normal (no ischemia) or Abnormal (evidence of ischemia). The Body Mass Index (BMI) was also extracted from the database for each patient. Concordance for the 2 tests was computed over a range of BMI values and graphically plotted for trend analysis. Patients were also grouped according to standard WHO bodyweight definitions: Normal (BMI < 25 kg/m2), overweight (BMI 25-30 kg/m2) and obese (BMI > 30 kg/m2) for statistical comparison of concordance.

## Results

Concordance for nuclear and cardiac stress perfusion imaging studies was 61.5% for BMI < 25 kg/m2, 33.3% for BMI 25-30 kg/m2, 33.3% for BMI 30-35 kg/m2 and 21.4% for BMI >35 kg/m2. A one-way ANOVA was computed to compare concordance between the 2 tests for patients categorized as Normal (BMI < 25), Overweight (BMI 25-30) and Obese (BMI > 30). Obese patients had a statistically significant decreased concordance between both tests when compared to normal weight patients (p <0.05).

## Conclusions

There is decreasing concordance between the results of nuclear stress perfusion imaging and MRI adenosine stress perfusion imaging with increasing bodyweight. Concordance is significantly decreased when comparing Obese vs Normal bodyweight patients. Cardiac MRI adenosine stress perfusion imaging appears to be a more appropriate diagnostic tool for obese patients.

## Funding

None.

